# Taking aim at Sox18

**DOI:** 10.7554/eLife.24238

**Published:** 2017-01-31

**Authors:** Injune Kim, Gou Young Koh

**Affiliations:** 1 Graduate School of Medical Science and Engineering, Korea Advanced Institute of Science and Technology, Daejeon, Republic of Korea; 1 Graduate School of Medical Science and Engineering, Korea Advanced Institute of Science and Technology, Daejeon, Republic of Koreagykoh@kaist.ac.kr; 2 Center for Vascular Research, Institute of Basic Science, Daejeon, Republic of Korea

**Keywords:** small molecules, transcription factors, protein-protein interactions, tumour angiogenesis, gene expression, Mouse, Zebrafish

## Abstract

A small molecule called Sm4 can disrupt interactions involving a transcription factor called Sox18, while having little impact on other members of the SoxF family.

**Related research article** Overman J, Fontaine F, Moustaqil M, Mittal D, Sierecki E, Sacilotto N, Zuegg J, Robertson AAB, Holmes K, Salim AA, Mamidyala S, Butler MS, Robinson AS, Lesieur E, Johnston W, Alexandrov K, Black BL, Hogan BM, De Val S, Capon RJ, Carroll JS, Bailey TL, Koopman P, Jauch R, Smyth MJ, Cooper MA, Gambin Y, Francois M. 2017. Pharmacological targeting of the transcription factor SOX18 delays breast cancer in mice. *eLife* **6**:e21221. doi: 10.7554/eLife.21221

Sox18 is a transcription factor that first came to prominence more than a decade ago when it was discovered that mutations in the gene for Sox18 cause HLTS, a rare human condition that involves loss of hair, build up of lymph fluid and vascular defects (Irrthum et al., 2003). Subsequent studies in zebrafish and mouse revealed that Sox18 is specifically expressed in the endothelial cells of blood vessels and is critical for several processes during the development of blood vessels (Sakamoto et al., 2007; Herpers et al., 2008); other studies showed that it also promotes the formation of new lymphatic vessels in embryonic mice (François et al., 2008).

However, Sox18 is also associated with cancer: in particular, the results of experiments on a mouse model of skin cancer suggest that it promotes a number of processes that help cancers to spread (Duong et al., 2012). Drugs that inhibit Sox18 could, therefore, help in the treatment of cancer. Now, in eLife, Mathias Francois of the University of Queensland and co-workers – including Jeroen Overman as first author – report that a small molecule called Sm4 inhibits Sox18 (Overman et al., 2017). Moreover, they went on to verify its anti-cancer effects and anti-metastatic effects in a mouse model of breast cancer. Sm4 is derived from a natural product that is found in the brown alga *C. cephalornithos*, and was identified by the Queensland-led collaboration in a high-throughput screen for potential Sox18 blockers (Fontaine et al., 2017).

Transcription factors are proteins that bind to specific DNA sequences (via a DNA binding domain) and control the rate at which genes are transcribed to produce molecules of messenger RNA. However, the targeting of transcription factors for therapeutic applications can be difficult. This is especially true for transcription factors that reside within the cell nucleus, such as Sox18, because the drug has to pass through the plasma membrane around the cell and then through the double-layered envelope around the nucleus. Drugs that target membrane proteins and cytoplasmic proteins, on the other hand, only have to pass through the plasma membrane.

Transcription factors also tend to be part of complex interaction networks, and targeting a single protein-protein interaction in the network is likely to have a relatively small impact, which makes it necessary to target a subset of the interactions in the network. Sox18, for example, is part of an interaction network that involves two other members of the SoxF family of transcription factors – Sox7 and Sox17 – as well as a number of other transcription factors (such as MEF2C, RBPJ, and OCT4).

Transcription factors can work alone or with other proteins, and some carry out their roles as dimers or trimers. Overman et al. – who are based at the University of Queensland and other institutes in Australia, China, the UK and the US – discovered that Sox18 works as a dimer in primary cultured endothelial cells: two Sox18 proteins can bind to each other to form a homodimer, or a single Sox18 protein can bind to another transcription factor (such as Sox7 or MEF2C) to form a heterodimer (Figure 1). Moreover, Overman et al. demonstrated that Sm4 disrupts a subset of interactions involving Sox18, but has only a modest impact on interactions involving Sox7 and Sox17. This means that Sm4 can repress the expression of the genes close to where Sox18 binds to DNA, but not those close to where other transcription factors bind to DNA.

**Figure 1.**
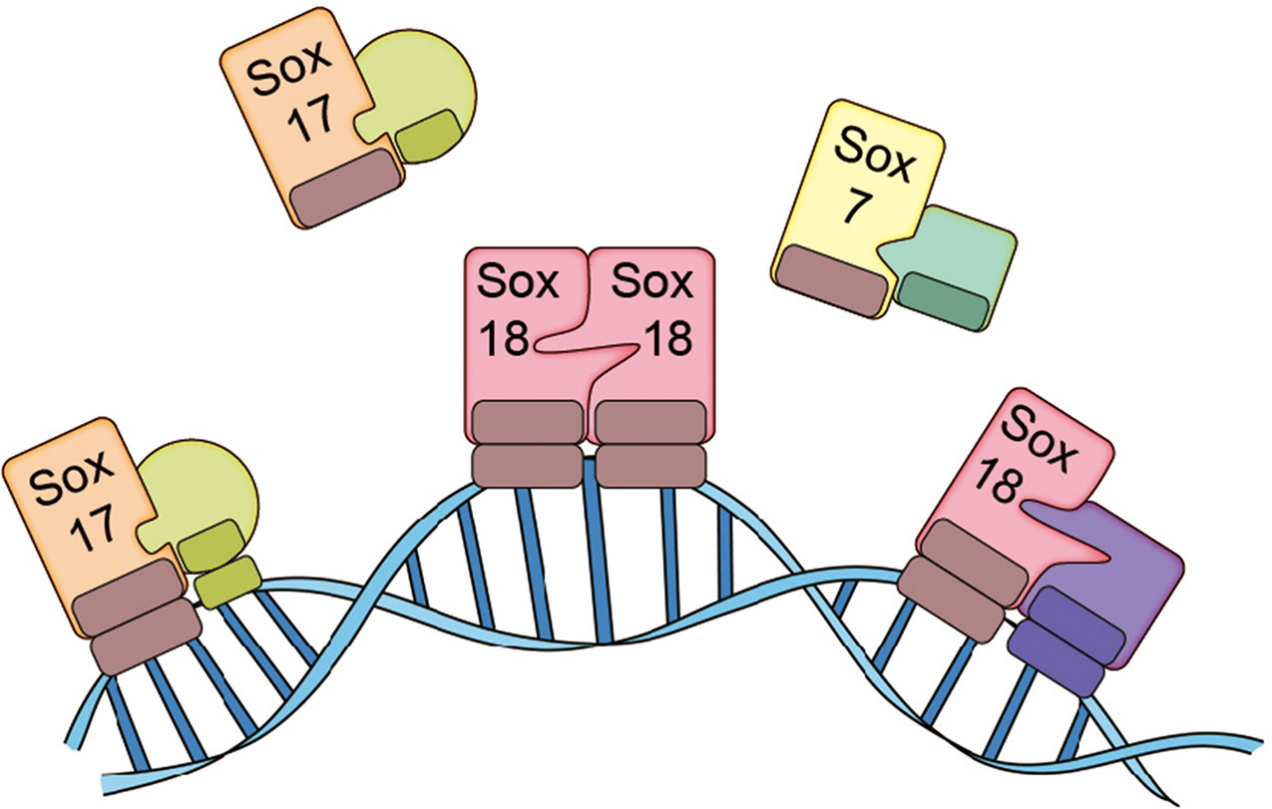
The SoxF family of transcription factors. Sox7, Sox17 and Sox18 all belong to the SoxF family of transcription factors and have identical DNA binding domains, so they bind to identical DNA motifs (Francois et al., 2010). However, by forming homodimers (such as Sox18-Sox18) or heterodimers (such as Sox7-RBPJ, Sox17-OCT4 or Sox18-MEF2C), they are able to bind to distinct regions of DNA because they need to recognize and bind to two consecutive DNA motifs. This allows the transcription factors to both co-operate and work on their own. Overman et al. show that a small molecule called Sm4 (not shown) can disrupt protein-protein interactions involving Sox18, while having little impact on those involving Sox7 and Sox17.

FIGURE CREDIT: Il-Kug Kim, Injune Kim, Gou Young Koh.

Neither Sox7 or Sox17 form homodimers: however, they both form heterodimers such as Sox7-RBPJ, Sox7-Sox18 or Sox17-OCT4. Since the expression of all members of the SoxF family appears to be restricted to endothelial cells, each family member can regulate some processes in blood and lymphatic vessels on its own, and regulate other process in tandem with other transcription factors, during both physiological and pathological conditions. In fact, some members of the family cooperate in developmental contexts (Kim et al., 2016), and act on their own in other contexts. It has been shown in mice, for example, that deficiency of the gene for Sox17 can induce intracranial aneurysm (Lee et al., 2015).

Overman et al. validate their in vitro findings in zebrafish larvae by demonstrating that Sm4 suppresses genes downstream of the gene for Sox18 and interferes with vascular development. They also showed that the anti-cancer and anti-metastatic effects of Sm4 in a mouse model of breast cancer were caused by the suppression of tumor lymphangiogenesis (the process by which tumors promote the formation of new lymphatic vessels) and the suppression of metastasis via the lymphatic system.

The work of Overman, Francois and co-workers elucidates the mechanism of Sox18 and provides a means to pharmacologically inhibit its function. However, there is still much that we do not know about the biological functions of the various transcription factors in the SoxF family: more advanced epigenetic and proteomic approaches using in vivo endothelial cells are needed to make progress in this area. Another challenge is the fact that patients with breast cancer often suffer from a secondary lymphedema, which occurs after removal of tumor tissues and their sentinel lymph nodes, so the risks of Sm4 or any other small molecule exacerbating this condition needs to be evaluated.
